# Postcoital Haemoptysis: A Case Report and a Review of the Literature

**DOI:** 10.1155/2013/189326

**Published:** 2013-05-08

**Authors:** Emeka B. Kesieme, Browne C. Okonkwo, Peter O. Okokhere, Georgi Prisadov, Eghosa Aigbe, Christopher Affusim

**Affiliations:** ^1^Department of Surgery, Irrua Specialist Teaching Hospital, PMB 8, Irrua, Edo State, Nigeria; ^2^Department of Medicine, Central Hospital Agbor, PMB 2005, Agbor, Delta State, Nigeria; ^3^Department of Medicine, Irrua Specialist Teaching Hospital, PMB 8, Irrua, Edo State, Nigeria; ^4^Department of Thoracic Surgery, Hospital Bremen-Ost, Osterholzer Landstraße 51, Bremen, Germany; ^5^Department of Family Medicine, Irrua Specialist Teaching Hospital, PMB 8, Irrua, Edo State, Nigeria

## Abstract

Haemoptysis is rarely reported following coitus, and cardiac decompensation has been mostly implicated in the aetiology. We present a 53-year-old Nigerian, known hypertensive diabetic woman with background ischaemic heart disease who presented with postcoital haemoptysis of one-year duration. Echocardiography revealed combined ischaemic and mitral valvular heart disease, probably of rheumatic aetiology. There has been no previous report in an African population. This case illustrates the need to rule out coitus as a rare but potential cause of haemoptysis in middle aged patients with underlying cardiac pathologies and the need for an extensive cardiac workup in a population with predominantly pulmonary causes of haemoptysis.

## 1. Introduction

Common causes of haemoptysis in sub-Saharan Africa and third world countries include tuberculosis, bronchiectasis, bronchogenic carcinoma, and pulmonary aspergilloma [1, 2]. 

Coitus, a form of isometric exercise, is largely tolerated despite an increased demand on the cardiovascular system. However exertion associated with sexual stimulation and coitus can cause transient haemoptysis due to a transient elevation of pulmonary capillary pressure with rupture of capillaries leading to haemoptysis [3]. 

We present a 53-year-old African, known hypertensive diabetic woman with ischaemic heart disease who presented with postcoital haemoptysis of one-year duration.

This is, to the best of our knowledge, the first reported case in an African and the only case implicating coexisting ischaemic and rheumatic valvular heart diseases as a cause of postcoital haemoptysis.

## 2. Case Study

A 53-year-old Nigerian, known hypertensive diabetic woman with a previous history of ischaemic heart disease presented with haemoptysis after coitus. This has been consistently observed for the past one year. There is no history of haemoptysis following any other physical or strenuous exercise, but she had an episode of haemoptysis following masturbation. There was no marked change in frequency of intercourse, sexual habit, or sexual partner.

There is a positive history of dyspnea on exertion, but no history of orthopnea or paroxysmal nocturnal dyspnea. Blood pressure has been well controlled with aldactone 25 mg dly, losartan 25 mg bd, and carvedilol 2.5 mg dly. She uses metformin 500 mg bd and glibenclamide 2.5 mg dly for the control of Diabetes Mellitus.

Physical examination was essentially normal. 

She had a flexible bronchoscopic examination of the upper airway. No mass lesion was seen but she desaturated speedily during the procedure.

Routine investigations (full blood count including platelet count, clotting profile, electrolytes, and urea and lipid profile) were normal.

Electrocardiography revealed moderate left axis deviation and left ventricular hypertrophy with repolarization abnormalities. Echocardiography revealed both systolic and diastolic dysfunctions and global hypokinesia of the left ventricular wall, worse in the interventricular septum. There is combined mitral stenosis and regurgitation probably from rheumatic valvular disease. Echocardiography done 2 years ago in another center before the onset of postcoital haemoptysis revealed a grossly dilated (L) ventricle with poor systolic contractility and segmental hypokinesia of the inferior wall. She has been referred to another center for cardiac catheterization and subsequently open cardiac surgery, as facilities for these procedures are presently not available in our center.

## 3. Discussion 

We did a literature search on the subject from 1900 till date using manual library search and journal publications on PubMed/Medline, Google Scholar, and EMBASE using the following keywords: postcoital haemoptysis, sex and haemoptysis, and haemoptysis during sexual intercourse. 

Ten (10) reports of postcoital haemoptysis have been published so far and 12 patients have been documented to have presented with postcoital haemoptysis. The age and sex of the patient were not documented in the report by Thompson [4]. Out of the remaining 11 patients, 6 were female while 5 were male. The age range is between 35 years and 81 years.

The reported causes of postcoital haemoptysis included hypertension [5, 6], left atrial tumour and mitral stenosis [4], left ventricular failure [7], pulmonary embolism [8], coronary artery disease [9], mitral regurgitation [10], amyloidosis [11], lymphangioleiomyomatosis and tuberous sclerosis [12], mitral regurgitation and pulmonary hypertension [13], Takayasu arteritis [6], and combined mitral stenosis and regurgitation from longstanding rheumatic heart disease in a known hypertensive patient [6]. The cause of postcoital haemoptysis in the index case report is combined ischaemic and rheumatic mitral valvular stenosis and incompetence. This supports the fact that postcoital haemoptysis results mainly from cardiac pathology. The only implicated pulmonary cause of postcoital haemoptysis is lymphangioleiomyomatosis, a condition characterized by smooth muscle proliferation around lymphatics, blood vessels, and alveolar airway [12].

Despite the fact that most postcoital haemoptysis result from cardiac pathology, bronchoscopy is indicated to rule out pulmonary causes. This is especially important in cases presenting in Africa and other third world countries. Half of the patients reported with postcoital haemoptysis had bronchoscopy [5–7, 11, 12]. During bronchoscopy of 2 reported cases with underlying hypertension, blood pressure elevation was observed during the procedure [5, 6]. We observed an unusual rapid desaturation during the bronchoscopy of the index patient.

Apart from Badawi and Geddes [12], who reported haemoptysis following coitus and after swimming, no other authors have reported any other physical activity as a cause of haemoptysis in a patient with postcoital haemoptysis.

Sexual activity places increased demands on the cardiovascular system, and its effects on the cardiovascular system differ significantly from other physical activities on account of its sympathetic activation [14]. In normal subjects, blood pressure and heart rate have been shown to increase during coitus just briefly, with peak coital blood pressure occurring at the onset of the plateau phase with subsequent normalization [15]. An increase in heart rate, right ventricular systolic and diastolic pressures, and pulmonary artery pressures was found to be significantly more during coitus when compared to usual forms of exercise in a patient with congestive cardiac failure and pulmonary hypertension [16]. Coitus may lead to a greater elevation in pulmonary venous pressure, increase in pulmonary capillary pressure, rupture of capillaries, and subsequent haemoptysis [3].

In the index case, haemoptysis resulted from a transient exacerbation of an already elevated pulmonary venous pressure following coitus on a background of ischaemic and mitral valvular disease with both ventricular systolic and diastolic dysfunctions.

## 4. Conclusion

Postcoital haemoptysis is rare and mainly results from cardiovascular compromise. There is a need for proper evaluation to determine the cause of haemoptysis, which will determine the proper line of management. Owing to the fact that cardiac pathologies are more likely to cause postcoital haemoptysis than pulmonary pathologies, it may be wise to initially investigate ruling out the cardiac causes.
